# ΔNp63 drives metastasis in breast cancer cells *via* PI3K/CD44v6 axis

**DOI:** 10.18632/oncotarget.11022

**Published:** 2016-08-02

**Authors:** Simone Di Franco, Alice Turdo, Antonina Benfante, Maria L. Colorito, Miriam Gaggianesi, Tiziana Apuzzo, Raju Kandimalla, Aurora Chinnici, Daniela Barcaroli, Laura Rosa Mangiapane, Giuseppe Pistone, Salvatore Vieni, Eliana Gulotta, Francesco Dieli, Jan Paul Medema, Giorgio Stassi, Vincenzo De Laurenzi, Matilde Todaro

**Affiliations:** ^1^ Department of Surgical and Oncological Sciences, Cellular and Molecular Pathophysiology Laboratory, University of Palermo, Palermo, Italy; ^2^ Dipartimento di Scienze Mediche, Orali e Biotecnologiche, University “G. d'Annunzio” Chieti-Pescara, CESI-MeT, Chieti, Italy; ^3^ Laboratory for Experimental Oncology and Radiology, Center for Experimental Molecular Medicine, Academic Medical Center, University of Amsterdam, Amsterdam, The Netherlands; ^4^ DISPUTer, University “G. d'Annunzio” Chieti-Pescara, Chieti, Italy; ^5^ Department of DIBIMIS, Dermatology Section, University of Palermo, Palermo, Italy; ^6^ Central Laboratory of Advanced Diagnosis and Biomedical Research (CLADIBIOR), University of Palermo, Palermo, Italy; ^7^ DiBiMIS, University of Palermo, Palermo, Italy

**Keywords:** breast cancer initiating cells, p63, PI3K/AKT pathway, CD44v6, metastasis

## Abstract

P63 is a transcription factor belonging to the family of p53, essential for the development and differentiation of epithelia. In recent years, it has become clear that altered expression of the different isoforms of this gene can play an important role in carcinogenesis. The *p63* gene encodes for two main isoforms known as TA and ΔN p63 with different functions. The role of these different isoforms in sustaining tumor progression and metastatic spreading however has not entirely been clarified.

Here we show that breast cancer initiating cells express ΔNp63 isoform that supports a more mesenchymal phenotype associated with a higher tumorigenic and metastatic potential. On the contrary, the majority of cells within the tumor appears to express predominantly TAp63 isoform. While ΔNp63 exerts its effects by regulating a PI3K/CD44v6 pathway, TAp63 modulates this pathway in an opposite fashion. As a result, tumorigenicity and invasive capacity of breast cancer cells is a balance of the two isoforms. Finally, we found that tumor microenvironmental cytokines significantly contribute to the establishment of breast cancer cell phenotype by positively regulating ΔNp63 and CD44v6 expression.

## INTRODUCTION

The development of epithelial tissue is a finely regulated process that involves several transcriptional factors [1]. p63, a p53 homolog, plays a key role in the generation of all the stratified squamous epithelia and their derivatives, including breast [2, 3].

The *p63* gene encodes six different isoforms, based on the presence of an amino-terminal trans-activation domain (*TAp63*) or its absence (*ΔNp63*), which can undergo alternative splicing at the 3′ end of the gene to generate the α, β and γ isoforms [4, 5]. Their structure suggests that TAp63 and ΔNp63 have distinct and opposing functions.

The role of p63 in stratified epithelium development was determined using murine genetic models. p63 KO mice showed alterations of all stratified epithelia as well as of epithelial appendages, including the mammary gland. The interpretation of these results about the role of p63 in epithelial development is still controversial and different models have been proposed. One model suggests that p63 is essential for the commitment and differentiation of epithelial precursors, while the other proposes that it is essential for the maintenance of the progenitor population [2, 3]. A third model attempts to reconcile the previous ones, suggesting that ΔNp63 is essential for the maintenance of the progenitor population while TAp63 is necessary to allow complete differentiation [6–9].

p63 has a major role in breast development, in the mature mammary gland it is confined to the early mammary progenitors and myoepithelial/basal cells, where ΔNp63 is the predominant isoform [10, 11]. ΔNp63 enhances cell proliferation [12] and inhibits apoptosis [13], whereas TAp63 induces apoptosis [14] and inhibits cell-cycle progression [15] leading to cell differentiation.

Current models describe healthy and cancer tissue growth as driven by stem cells. In this context, genetic/epigenetic changes that affect normal stem cells can lead to production and expansion of cancer initiating cells (CICs) [16, 17], which have been isolated and characterized in many solid tumors, including breast [18, 19]. Recent research has shown that the origin of normal and cancer stem cells in breast tissue may also be caused by de-differentiation of committed healthy and cancer cells, thus making the cellular heterogeneity and hierarchy more complex [20]. This plasticity seems to be regulated by the microenvironment and mainly through the induction of an EMT programme. It was also recently demonstrated that breast CICs (BCICs) exist in distinct mesenchymal- and epithelial-like states that are characterized by specific markers and gene-expression profiles [21]. Interestingly, the mesenchymal-like BCICs have a similar gene-expression profile to basal stem cells, whereas the epithelial-like BCICs show luminal stem cell traits that are found in normal breast tissue.

CD44v6 has recently been described as both a functional biomarker and a therapeutic target of metastatic CICs, through the activation of PI3K signaling [22]. Of note, the PI3K pathway has been demonstrated to play an important function in the development of breast cancer, as it is deregulated in almost 50% of patients [23]. For these reasons, PI3K pathway is considered an attractive biological target, to be mainly applied in the treatment of the most aggressive tumors [24].

Here we show that breast cancer sphere cells (BCSCs), known to be enriched in cells with cancer initiating/stem-like features [16], are characterized by the expression of ΔNp63, which is crucial for the induction of an EMT programme, thus increasing the ability of breast cancer cells to migrate and form distant metastasis, through the activation of PI3K/AKT pathway and CD44v6 expression. Differently, cells bearing the TAp63 expression are endowed with tumorigenic potential but unable to form metastasis and tumors after serial transplantation, showing reduced levels of PI3K/AKT pathway activation. The selective targeting of CD44v6 led to a decrease of BCSCs proliferation, clonogenicity and invasion *in vitro*. Finally, tumor microenvironmental stimuli played a key role in the promotion of the BCSC phenotype that regulates the expression levels of ΔNp63 and CD44v6.

## RESULTS

### BCSCs with high tumorigenic and metastatic potential express higher levels of ΔNp63

**Figure 1:**
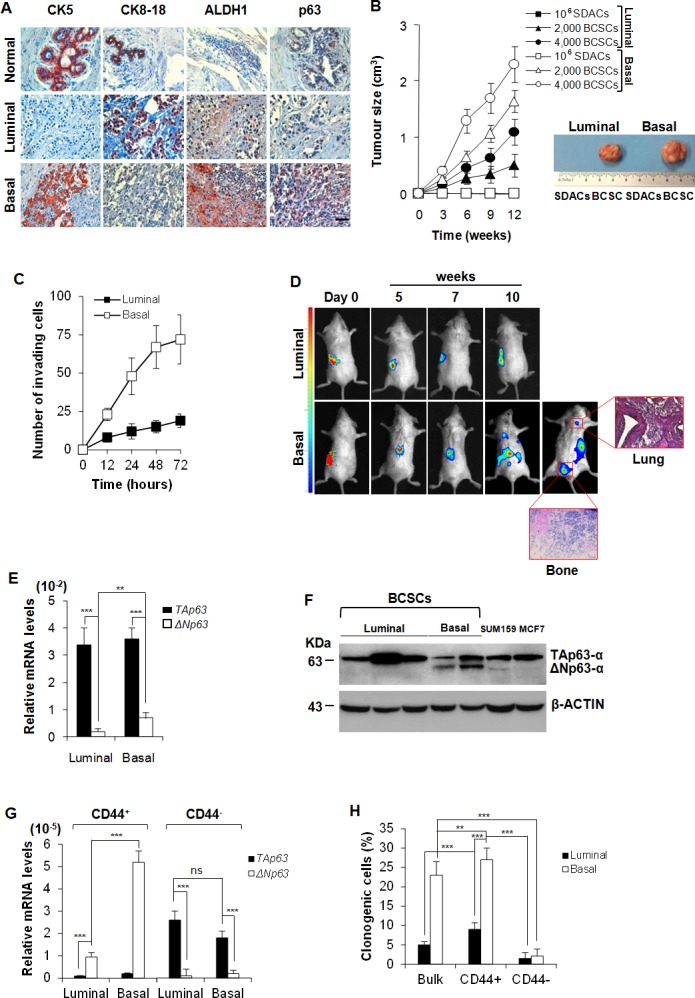
Basal BCSCs express ΔNp63 and retain a metastatic activity **A.** Representative immunohistochemical analysis for CK5, CK8-18, ALDH1 and p63 performed on paraffin embedded sections of 21 breast cancer clinical samples (15 luminal and 6 basal breast cancers) and 6 normal tissues. Scale bar represents 50 μm. **B.** Size of subcutaneous tumors generated by the injection of 10^6^ SDACs, and 2,000 or 4,000 luminal and basal BCSCs, derived from the same patient. Data are mean ± SD of 3 independent experiments, performed with cells from 2 luminal and 2 basal different cancer patients. (Right panel) Representative gross morphology of tumors outgrowth derived by implantation of SDACs or 4,000 BCSCs derived from the same patient at 12 weeks. **C.** Invasion assay of 2,000 luminal and basal BCSCs cultured in SFM up to 72 hours. Results are shown as mean ± S.D. of 3 independent experiments performed in 3 luminal and 2 basal different primary cell lines. **D.** *In vivo* whole-body imaging analysis of sub-renal capsule tumors and metastasis growth at the indicated time points. Xenografts were generated injecting 4,000 BCSCs into the sub-renal capsule. Data are representative of 16 tumor xenografts generated by the injection of 2 luminal and 2 basal BCSCs derived from different patients. (Red boxes) Representative H&E analysis of lung and bone metastasis generated by injection of basal BCSCs at 10 weeks. **E.** *TAp63* and *ΔNp63* mRNA expression levels in freshly purified luminal and basal breast cancer cells. *GAPDH* amplification was used as endogenous control. Results show mean ± S.D. of 3 independent experiments using 5 luminal and 5 basal cancer samples. **F.** Immunoblot analysis for TAp63- and ΔNp63-α in luminal and basal BCSCs. SUM159 and MCF7 cell lines were used as basal and luminal control, respectively. Data are representative of 3 independent experiments using 5 BCSC lines derived from 3 luminal and 2 basal breast cancer patients. **G.** mRNA expression levels of *TAp63* and *ΔNp63* in enriched CD44^+^ and CD44^−^ luminal and basal BCSCs. Data are mean ± S.D. of 3 independent experiments. (H) Clonogenic assay of bulk BCSCs, CD44^+^ and CD44^−^ enriched luminal and basal BCSCs. Data are expressed as mean ± S.D. of 3 independent experiments performed with BCSCs purified from 3 luminal and 2 basal cancer patients. * indicates *P* < 0.05, ** indicate *P* < 0.01 and *** indicate *P* < 0.001. ns indicates non statistically significant.

We freshly isolated breast cancer cells from surgical breast cancer resections. As detailed in the material and methods section, we cultured them either as sphere cells (Suppl. Figure 1A), referred to as BCSCs, or as sphere derived adherent cells (SDACs) (Suppl. Figure 1A). In order to establish the luminal and basal phenotype of our collection of breast cancer samples, we assessed the expression of clinical markers associated with specific breast cancer molecular subtypes [25–27] in formalin-embedded histological samples. In accordance with the well established characterization of breast carcinoma subtypes [18, 25, 26], luminal breast cancer samples were characterized by CK8-18^+^, CK5^−^, CK14^−^, ER^+^, PR^+/−^, HER2^+/−^, ALDH1^−^, while basal breast carcinomas were CK8-18^−^, CK5^+^, CK14^+^, ER^−^, PR^−^, HER2^−^, ALDH1^+^ [25] (Table 1, Figure 1A). Interestingly and in line with previous findings [28], basal breast cancers and their derived BCSCs showed higher expression of p63, as compared to the luminal samples (Figure 1A). Moreover, basal BCSCs retained higher levels of CK5, CK14, CD10 and VIMENTIN (Suppl. Figure 1B-1C).

**Table 1:** Breast cancer case description

Sample	Age	Tumor type	Grading	ER	PR	HER-2/neu	Ki67	p63	Mammosphere formation	Xenograft
**Pt #1**	47	IDC	G2	+	+	-	25%	+	No	-
**Pt #2**	65	IDC	G3	-	-	+++	>10%	++	No	-
**Pt #3**	63	IDC	G2	+	+	-	20%	+	No	-
**Pt #4**	55	IDC	G2	+	+	+++	>10%	+	Yes	7/10
**Pt #5**	85	IDC	G2	-	-	++	15%	+	No	-
**Pt #6**	57	IDC	G2	+	-	+	20%	+	No	-
**Pt #7**	46	IDC	G3	+	+	++	15%	+	Yes	4/10
**Pt #8**	68	IDC	G3	+	+	+	20%	+	No	-
**Pt #9**	86	IDC	G2	+	+	++	15%	+	No	-
**Pt #10**	85	ILC	G2	-	-	+	>10%	++	Yes	10/10
**Pt #11**	70	IDC	G2	+	+	-	10%	+	No	-
**Pt #12**	59	ILC	G2	+	+	-	<10%	+	No	-
**Pt #13**	69	IDC	G3	+	+	++	10%	+	No	-
**Pt #14**	53	IDC	G2	+	+	-	5%	+	Yes	ND
**Pt #15**	83	IDC	G3	-	+	+++	10	++	Yes	-
**Pt #16**	67	IDC	G3	+	+	-	10%	-	No	-
**Pt #17**	56	IDC	G2	+	+	++	10%	+	No	-
**Pt #18**	74	IDC	G2	+	+	+++	>10%	+	Yes	6/10
**Pt #19**	52	IDC	G3	+	+	-	10%	+	No	-
**Pt #20**	53	IDC	G2	+	+	+	10%	+	No	-
**Pt #21**	71	IDC	G3	+	+	++	25%	++	No	-
**Pt #22**	69	IDC	G2	+	+	+	25%	+	Yes	3/10
**Pt #23**	85	IDC	G2	+	+	-	15%	+	No	-
**Pt #24**	69	IDC	G2	+	+	-	>10%	+	Yes	-
**Pt #25**	77	IDC	G2	+	+	-	20%	+	No	-
**Pt #26**	64	IDC	G2	+	+	++	25%	+	No	-
**Pt #27**	73	IDC	G3	+	+	+	20%	+	No	-
**Pt #28**	80	IDC	G3	+	+	-	15%	+	No	-
**Pt #29**	71	IDC	G3	+	+	+	2%	+	No	-
**Pt #30**	52	IDC	G3	-	-	-	>10%	+++	Yes	10/10
**Pt #31**	81	IDC	G2	+	+	++	30%	++	No	-
**Pt #32**	67	IDC	G2	+	+	-	10%	+	No	-
**Pt #33**	63	IDC	G2	+	+	-	<10%	+	No	-
**Pt #34**	75	IDC	G2	+	+	-	35%	++	No	-
**Pt #35**	86	IDC	G3	+	-	++	15%	+	No	-
**Pt #36**	48	IDC	G3	+	+	+++	35%	+	No	-
**Pt #37**	68	IDC	G3	+	+	++	15%	+	No	-
**Pt #38**	76	IDC	G3	+	+	++	30%	+	No	-
**Pt #39**	65	IDC	G3	-	-	+++	80%	++	Yes	ND
**Pt #40**	53	IDC	G2	+	+	-	5%	+	Yes	ND
**Pt #41**	76	IDC	G2	+	+	-	10%	+	No	-
**Pt #42**	61	IDC	G2	+	+	-	45%	+	No	-

IDC: Infiltrating Ductal Carcinoma; ILC: Infiltrating Lobular Carcinoma; ND Not Detected

Although both luminal and basal BCSCs were capable of forming tumors when subcutaneously injected in NOD/SCID mice, basal BCSCs showed an enhanced tumorigenic potential than luminal ones (Figure 1B). The tumorigenic potential was lost upon deprivation of EGF and b-FGF followed by exposure to serum (SDACs), as shown by failure to form tumors despite the injection of high number (10^6^) of SDACs (Figure 1B).

Both basal BCSC primary lines derived from patient #10 and #30 retained *in vitro* invasive capacity and *in vivo* metastatic ability when injected under the renal capsule of immunodeficient mice (Figure 1C-1D). In contrast, luminal BCSCs were less invasive *in vitro* and generated only small primary tumors and were deficient for metastatic spread to lung and bone *in vivo* (Figure 1D).

We then further investigated mRNA expression of the different *p63* isoforms in our samples finding that both luminal and basal breast cancers display higher levels of *TAp63* than *ΔNp63*, and that *ΔNp63* levels are significantly higher in basal than luminal cancer subtypes (Figure 1E). Protein analysis confirms that while luminal cells express mainly the TA isoform (indeed ΔNp63 is undetectable) basal cell lines express both TA and ΔN isoforms (Figure 1F).

We then enriched the cancer initiating population by cell sorting based on CD44/CD24 expression (Suppl. Figure 1D), and evaluated the expression levels of the p63 isoforms [21]. CD44^+^ cells, which present the characteristics of the acronym CICs, express higher mRNA levels of *ΔNp63* than *TAp63* despite their luminal or basal origin. Conversely, CD44^−^ breast cancer compartment shows negligible mRNA levels of *ΔNp63* and high *TAp63* levels (Figure 1G). As expected, CD44^+^ BCSCs showed a higher clonogenic potential than CD44^−^ BCSCs (Figure 1H).

In line with these findings, cells grown as SDACs showed a gradual increase of TAp63 expression levels and a decrease of ΔNp63 levels as compared to cells grown as spheres (Suppl. Figure 1E). According to previous observations, we found that the exposure to serum dramatically reduced VIMENTIN and increased MUC1, while slightely diminished CD10 expression in basal SDACs (Suppl. Figure 1F), compared to BCSCs (Suppl. Figure 1B-1C), confirming a less stem-like phenotype in these culture conditions. These results suggest that ΔNp63 may play a role in driving the tumorigenic and metastatic capacity of CD44 enriched BCSCs.

### ΔNp63 over-expression results in a more aggressive phenotype

**Figure 2:**
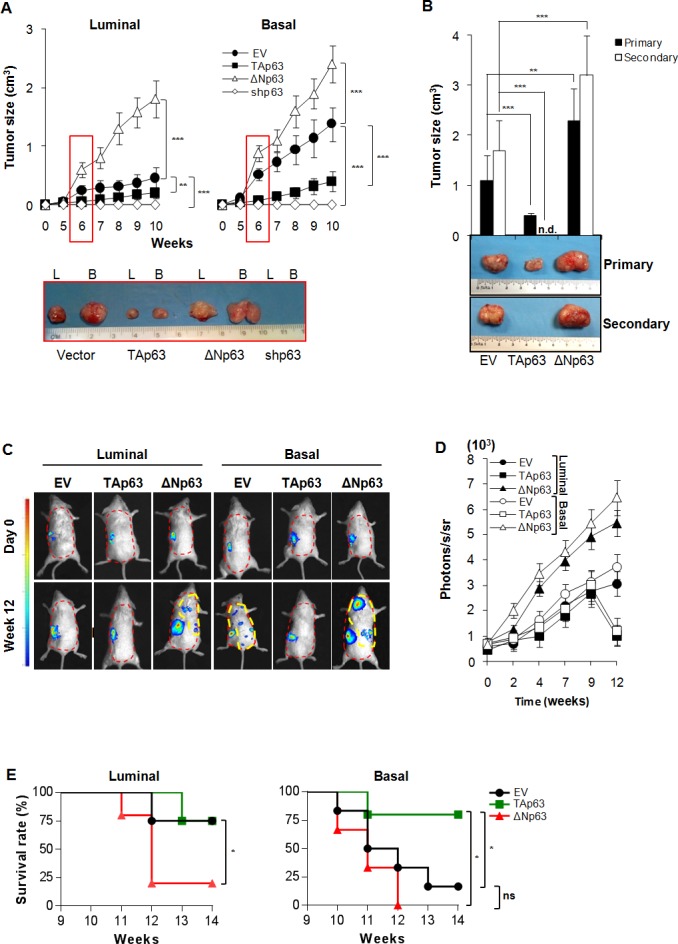
ΔNp63 confers metastatic potential to BCSCs **A** Size of subcutaneous tumors following injection of 4,000 luminal and basal BCSCs overexpressing TAp63, ΔNp63, or shp63 (empty vector, EV, was used as control). Data are represented as mean ± SD of 8 tumors generated by the injection of 2 luminal and 2 basal BCSCs derived from different patients. (Lower panel) Representative gross morphology of luminal (L) and basal **B** tumor xenografts at 6 weeks. **B** Size of primary (9 weeks) and secondary (6 weeks) serial transplantation of subcutaneous tumor xenografts obtained by injection of 4,000 empty vector (EV), TAp63 and ΔNp63 derived from 2 basal BCSC primary lines. n.d. indicates: no detected tumors. **C** *In vivo* imaging analysis of sub-renal capsule xenograft tumors and metastasis growth (red dashed area) at the indicated weeks derived from the injection of 4,000 luminal and basal BCSCs transduced with empty vector (EV), TAp63 and ΔNp63. **D** *In vivo* imaging kinetics analysis as in **C** at the indicated time points. Data represent mean ± SD of 3 independent experiments performed with 2 luminal and 2 basal BCSCs derived from different patients. **E** Survival rate percentage of mice treated as in **C**, at the indicated weeks. Results were analyzed using the log-rank test. Photons/seconds/steradian (photons/s/sr) = 4 for all the metastatic lesions (yellow dashed area) was chosen as limit to proceed with mice sacrifice. * indicates *P* < 0.05, ** indicate *P* < 0.01 and *** indicate *P* < 0.001. ns indicates non statistically significant.

To further investigate the involvement of ΔNp63 in tumorigenic potential we used lentiviral vectors to over-express TAp63, ΔNp63 or silence all p63 isoforms using shRNA in both basal and luminal-derived BCSCs (Suppl. Figure 2A-2B). These cells were then injected subcutaneously into immunocompromised mice to test their ability to form tumors. Over-expression of ΔNp63 resulted in increased tumor growth rate while over-expression of TAp63 reduced it in both cell types (Figure 2A). Interestingly, silencing of both isoforms completely abrogated the BCSCs ability to form tumors *in vivo* (Figure 2A).

Moreover, TAp63 over-expression appeared to alter BCSCs behavior allowing them to adhere in serum free medium (SFM) conditions where they normally form spheres (Suppl. Figure 2C).

In order to confirm whether p63 isoforms could regulate self-renewal potential, basal BCSCs over-expressing TAp63 and ΔNp63 were serially subcutaneously transplanted in NOD/SCID mice. At the second passage, ΔNp63 over-expressing cells maintain the ability to promote the formation of tumors, whose size was significantly larger than the control. Conversely, cells over-expressing TAp63 lacked the ability to establish tumors as secondary xenografts (Figure 2B).

We then investigated the role of TAp63 and ΔNp63 in influencing the metastatic potential of BCSCs [29, 30]. Cells over-expressing p63 isoforms were allowed to grow in the sub-renal capsule in immunocompromised mice for up to 12 weeks. We found that TAp63 reduced the ability of basal BCSCs to give rise to metastasis, whereas luminal cells over-expressing ΔNp63 gained the capacity to engraft the kidney and metastasize to distant organs such as lung (Figure 2C-2D and Suppl. Figure 2D). To ensure that metastases were generated by the distant colonization of human TAp63 and ΔNp63 BCSCs, previously injected in mice sub-renal capsule, we performed immunohistochemical stainings for human CK-AE (Suppl. Figure 2E). As expected, ΔNp63 over-expression reduced survival rate of mice while TAp63 increased it as compared to controls (Figure 2E). All these data show that ΔNp63 supports a more aggressive phenotype regardless the origin of the BCSCs, inducing increased tumor formation ability and metastatic spreading.

### p63 isoforms regulate EMT traits

**Figure 3:**
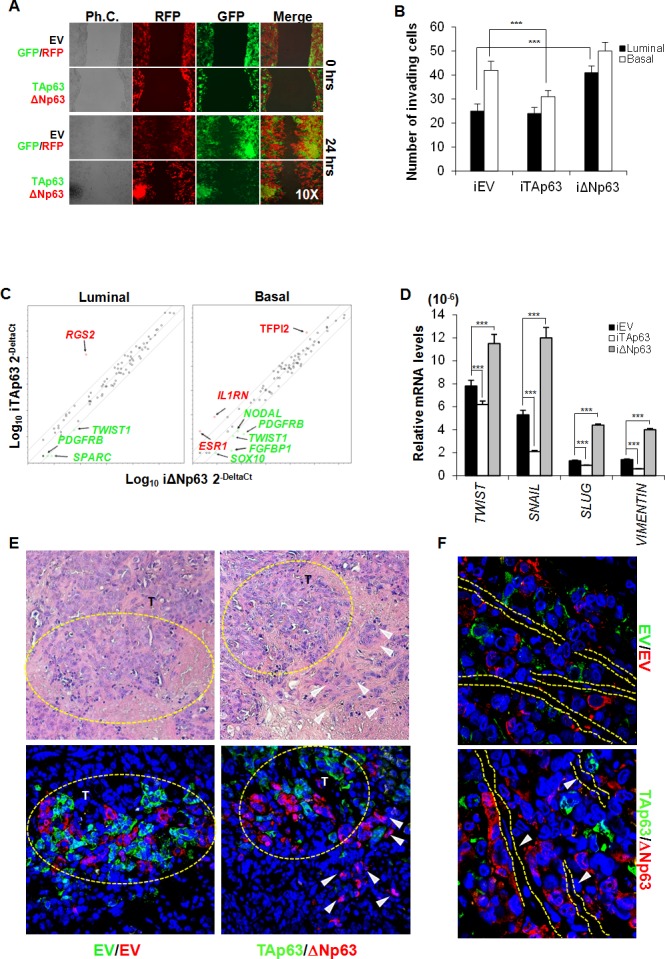
EMT program is regulated by ΔNp63 expression **A.** Representative wound healing analysis of 3 independent experiments performed with 3 luminal BCSCs over-expressing TAp63-GFP (green color) or ΔNp63-RFP (red color). Cell migration was monitored for 24 h. **B.** Invasion assay performed at 72 hours with luminal and basal iEV, iTAp63 or iΔNp63 BCSCs following 5 days of exposure to doxycycline. Data are represented as mean ± S.D. of 3 independent experiments derived from 3 luminal and 2 basal BCSC lines. **C.** EMT-related gene expression analysis of luminal and basal iEV, iTAp63 or iΔNp63 BCSCs following 3 days of exposure to doxycycline. Fold variation of ΔNp63 *vs* TAp63 ≥ 3 are shown in green or red. **D.** *TWIST, SNAIL, SLUG* and *VIMENTIN* mRNA expression levels in luminal BCSCs as in **C.** Results show mean ± S.D. of 3 independent experiments. **E.** (Upper panels) Representative H&E staining performed on paraffin-embedded sections of xenografts generated by subcutaneous injection of 1:1 mixture of EV-GFP:EV-RFP or TAp63-GFP:ΔNp63-RFP luminal BCSCs. (Lower panels) Immunofluorescence analysis of TAp63-GFP (green color) or ΔNp63-RFP (red color) performed on paraffin-embedded sections of xenografts generated as in **E** Dashed yellow circles represent tumor growth areas (T). White arrowheads indicate tumor buds overexpressing ΔNp63-RFP (red color) at the tumor invasion front. **F.** Representative immunofluorescence analysis of TAp63-GFP (green color) or ΔNp63-RFP (red color) performed on paraffin-embedded sections of xenografts generated as in **E** White arrowheads and dashed lines indicate vessels. * indicates *P* < 0.05, ** indicate *P* < 0.01 and *** indicate *P* < 0.001. ns indicates non statistically significant.

We tested the migration capacity of BCSCs over-expressing either TAp63 or ΔNp63 using a wound healing assay, which was performed using luminal BCSCs transduced with TAp63-GFP or ΔNp63-RFP lentiviral vectors (or with empty vectors EV-GFP and EV-RFP). By mixing an equal number of TAp63-GFP and ΔNp63-RFP or EV-GFP and EV-RFP cells, after 24 hours we observed that, while in the control setting GFP and RFP cells are randomly interspersed, uniquely ΔNp63 BCSC are confined to the migratory edge compared to TAp63 BCSCs (Figure 3A). Since a prolonged expression of high TAp63 levels could determine cell death induction, we used inducible vectors encoding for p63 isoforms (iEV, iTAp63 and iΔNp63). Similar results were obtained performing an invasion assay, which showed that the high expression of ΔNp63 in luminal BCSCs is correlated with an enhanced invasive capacity (Figure 3B) paralleled by an increase of CD44^+^/CD24^−^ (Suppl. Figure 2F). Conversely, expression of TAp63 in basal BCSCs significantly hampered their ability to invade (Figure 3B), reducing the mesenchymal-like CD44^+^/CD24^−^ cell compartment (Suppl. Figure 2F). ALDH activity showed no significant variations (Suppl. Figure 2F).

In order to explore which signaling pathways involved in the metastatic process of BCSCs are regulated by p63, we analyzed the modifications of EMT-related genes by gene array analysis. While three days of ΔNp63 induction generally up-regulated most of the EMT-related genes, TAp63 induction reduced the expression levels of these genes in both luminal and basal BCSCs (Figure 3C). Interestingly, both luminal and basal BCSCs over-expressing ΔNp63 showed high levels of *PDGFRB* and *TWIST1* (Figure 3C). Moreover, in basal BCSCs, ΔNp63 down-regulated *TFPI2* (> 5 fold regulation), *IL1RN* (> 10 fold regulation), and *ESR1* (> 10 fold regulation) [31] and increased the mRNA expression levels of *SOX10* (> 12 fold regulation), *NODAL* (> 3 fold regulation), and *FGFBP1* (> 3 fold regulation) (Figure 3C). qRT-PCR confirmed that ΔNp63 significantly increased the mRNA levels of the crucial EMT-related genes *TWIST*, *SNAIL*, *SLUG* and *VIMENTIN* (Figure 3D). Though luminal and basal BCSCs showed different combinations of activated/repressed transcription factors and signaling pathways, these data indicate that the induction of different p63 isoforms influences their migratory/invasive behaviour. Finally, using luminal BCSCs transduced with TAp63-GFP or ΔNp63-RFP lentiviral vectors we tested *in vivo* localization of TAp63 and ΔNp63 cells in tumors. Interestingly, xenografts generated by the injection of a 1:1 ratio of TAp63-GFP/ΔNp63-RFP luminal BCSCs showed that cells over-expressing ΔNp63 were located as tumor buds at the invasive front (Figure 3E). Strikingly, ΔNp63 over-expressing cells were localized in close proximity to endothelial cells suggesting their migratory aptitude (Figure 3F). Hence, ΔNp63 might play a key role in EMT induction and in the invasive potential of BCSCs under the influence of tumor microenvironment.

### ΔNp63 induces CD44v6 expression

**Figure 4:**
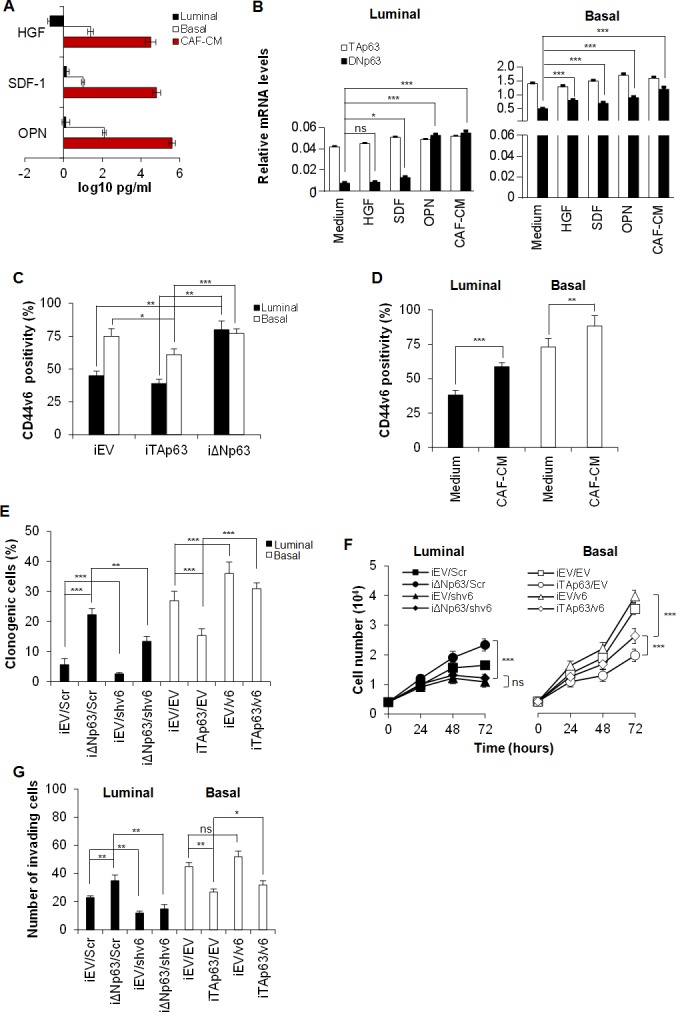
ΔNp63 enhances CD44v6 expression **A.** HGF, SDF-1 and OPN levels in supernatants of luminal and basal BCSCs, and cancer associated fibroblasts (CAFs) freshly isolated from a basal breast tumor sample. Data are mean ± SD of 3 independent experiments performed with 3 luminal and 2 basal BCSC lines derived from different patients. **B.** *TAp63* and *ΔNp63* mRNA expression levels in luminal and basal BCSCs, exposed to HGF, SDF-1 and OPN or CAF-conditioned medium (CAF-CM) for 24 hours. *GAPDH* amplification was used as endogenous control. Results show mean ± SD of 3 independent experiments performed with 3 luminal and 2 basal BCSC primary lines. **C.** Percentage of CD44v6 positive cells in luminal and basal iEV, iTAp63 or iΔNp63 BCSCs performed by flow cytometry. Data are shown as mean ± SD of 3 independent experiments using 3 luminal and 2 basal BCSC primary lines. **D.** CD44v6 positivity percentage in luminal and basal BCSCs treated with medium (Medium) or CAF-CM for 24 hours. Results are mean ± SD of 3 independent experiments accomplished using 3 luminal and 2 basal BCSCs derived from different patients. **E.** Percentage of clonogenic luminal BCSCs transduced with either or both iΔNp63 and shCD44v6 (shv6) vector and their control, iEV and Scr respectively. Basal BCSCs were transduced with either or both iTAp63 and CD44v6 (v6) expressing vectors and their iEV/EV controls. BCSCs were firstly transduced with the inducible isoforms of p63 and after one week with shv6 and v6. After 7 days, cells were exposed to doxycycline for 5 days before the experiment and plated in doxycycline containing medium for the entire assay. Results are mean ± SD of 3 independent experiments performed with 3 luminal and 2 basal BCSC primary lines. **F.** Cell number analysis of luminal and basal BCSCs as in **E**, up to 72 hours. **G.** Invasion assay at 72 hours of luminal and basal BCSCs treated as in **E.** Results are mean ± SD of 4 independent experiments. * indicates *P* < 0.05, ** indicate *P* < 0.01 and *** indicate *P* < 0.001. ns indicates non statistically significant.

Tumor associated cytokines are known to enhance cell migration and invasion [32–35]. By analyzing the most common tumor microenvironmental cytokines, we observed that basal BCSCs, as well as cancer-associated fibroblasts (CAFs) isolated from a basal cancer sample, secrete hepatocyte growth factor (HGF), stromal cell-derived factor 1 (SDF-1) and osteopontin (OPN) in the medium (Figure 4A). Exposure to SDF-1, OPN, or CAF-conditioned medium (CAF-CM) markedly increased the expression levels of *ΔNp63* in both luminal and basal BCSCs (Figure 4B). ΔNp63 regulates the expression of CD44 splicing isoforms in head and neck cancer [32]. Moreover, we have recently demonstrated that tumor niche is an extraordinary source of microenvironmental stimuli that induce CD44v6 expression, leading to the reprogramming of CD44v6^−^ progenitors in metastatic CD44v6^+^ stem cells [22]. In this context, we explored whether p63 isoforms could regulate CD44 isoforms expression also in breast cancer. Indeed, CD44 has been amply reported as a marker that identifies breast cancer cells with a pronounced self-renewal and metastatic potential [33] and, interestingly, circulating tumor cells endowed with metastatic capabilities have been shown to highly express the CD44 isoform variant, CD44v6 [34].

To investigate if CD44v6 has a functional role on BCSCs and whether it is differently modulated by p63 isoforms, we firstly tested CD44v6 expression on iTA/iΔNp63 BCSCs. iΔNp63 BCSCs express significantly higher CD44v6 levels than iTAp63 BCSCs (Figure 4C). Moreover, the over-expression of ΔNp63 in luminal BCSCs enforced CD44v6 expression while it was constant in basal BCSCs, which already harbour high level of CD44v6 (Figure 4C).

In colorectal progenitor cells, CD44v6 is boosted by the secretion of OPN, SDF-1, HGF microenvironmental cytokines [22]. Thus, we tested whether CAF-CM could affect CD44v6 expression in BCSCs. We found that luminal BCSCs treated with CAF-CM increased CD44v6 expression levels (Figure 4D), likely for an increase in ΔNp63 isoform.

To demonstrate the presence of a functional link between p63 isoforms and CD44v6 in BCSC behaviour, we performed a clonogenic, proliferation and invasion assay on iTAp63 basal and iΔNp63 luminal BCSCs transduced with lentiviral vectors encoding for CD44v6 (v6) or shCD44v6 (shv6), respectively. Since luminal BCSCs harbor lower levels of ΔNp63 than basal ones (Figure 1F), CD44v6 was down-regulated in luminal BCSCs over-expressing ΔNp63 (iΔNp63/shv6) and up-regulated in the basal subtype over-expressing TAp63 (iTAp63/v6) (Figure 4E-4G). In brief, BCSCs were initially transduced with iEV, iTAP63 or iΔNp63, and after 7 days with vectors coding for v6 or shv6 and their controls, EV and Scr respectively. After one week, cells were exposed to doxycycline for 5 days (to induce expression of the p63 isoforms). While the down-regulation of CD44v6 blunted the clonogenic, proliferative and invasive capability of luminal BCSCs bearing iΔNp63, over-expression of CD44v6 restored these features in basal BCSCs transduced with iTAp63 (Figure 4E-4G).

### ΔNp63 enhances PI3K/AKT pathway activity controlling BCSCs chemoresistance

**Figure 5:**
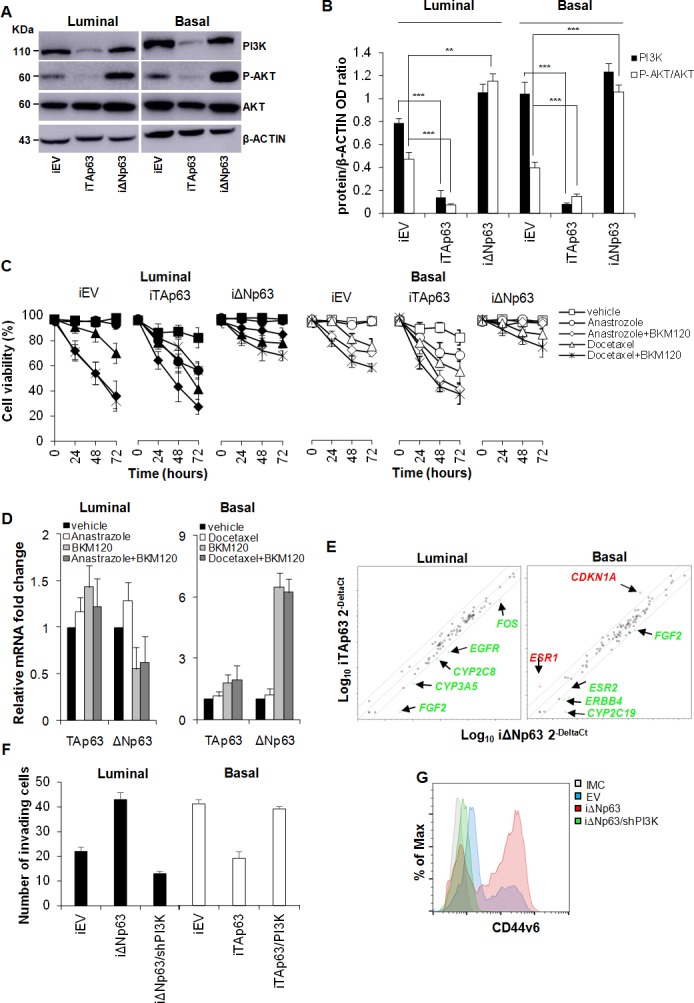
TAp63 sensitizes BCSCs to therapeutic drugs **A.** Immunoblot analysis of PI3K, P-AKT and AKT, in luminal and basal BCSCs transduced with doxycycline inducible EV, TAp63 or ΔNp63 (iEV, iTAp63 and iΔNp63). β-ACTIN was used as loading control. **B.** Relative expression levels of PI3K and P-AKT/AKT as in **A.** Data are expressed as mean ± SD of 3 independent experiments. **C.** Cell viability analysis of BCSCs transduced as in **A** and treated with Anastrozole or Docetaxel, alone or in combination with BKM120 up to 72 hours. Data are mean ± SD of 3 independent experiments. **D.** Relative TAp63 and ΔNp63 expression in luminal and basal BCSCs that survived to the above mentioned treatments. Data are expressed as mean ± SD of 3 independent experiments performed in 3 luminal and 2 basal BCSC lines. **E.** Scatter plot of drug-resistance related genes in luminal and basal BCSCs as in **A.** Genes indicated in green and red show fold variation (ΔNp63 *vs* TAp63) ≥ 3. **F.** Invasion assay of luminal iEV, iΔNp63 or iΔNp63/shPI3K and basal iEV, iTAp63 or iTAp63/PI3K performed at 72 hours. Data are represented as mean ± SD of 3 independent experiments using 3 luminal and 3 basal BCSCs. **G.** Flow cytometry analysis of CD44v6 in luminal BCSCs as in **F.** * indicates *P* < 0.05, ** indicate *P* < 0.01 and *** indicate *P* < 0.001. ns indicates non statistically significant.

CD44v6 is essential for colorectal CSC migration and metastatic outgrowth and its expression is sustained by PI3K/AKT pathway [22]. Indeed, PI3K is involved in multiple cellular events and is required for proliferation and migration of different cancer cell types including breast cancer cells [22, 35]. Starting from the observation that in our model CD44v6 expression is augmented in luminal BCSCs following the over-expression of ΔNp63 (Figure 4C) we sought to explore whether p63 could modulate the activation of the PI3K/AKT pathway. Western Blot analyses showed that exogenous expression of TAp63 reduced PI3K/AKT pathway activation, as shown by a considerable decrease in AKT phosphorylation (Figure 5A-5B). Conversely, ΔNp63 over-expression strongly boosted this signalling pathway (Figure 5A-5B). Notably, whereas a similar reduction in p-AKT expression level is observed in luminal and basal TAp63 over-expressing cells, over-expression of ΔNp63 led to a more pronounced phosphorylation of AKT in basal cells as compared to luminal ones (Figure 5A-5B). In line with the observation that ΔNp63 could promote a proliferative phenotype through the regulation of the PI3K/AKT pathway, we administered *in vitro* the PI3K inhibitor (BKM120) in combination with either a non-steroidal aromatase inhibitor (Anastrozole) or a member of the taxane drug class (Docetaxel) (Figure 5C). Indeed, aromatase inhibitors in combination with PI3K inhibitors are currently undergoing clinical trials in order to overcome refractoriness to standard anti-tumoral therapies in estrogen receptor-positive (luminal) breast cancers [36]. Moreover, PI3K inhibitors in combination with taxanes has shown to enforce the effectiveness of taxanes in both estrogen receptor-positive and -negative breast cancers [37].

Interestingly, iΔNp63 rendered luminal BCSCs more refractory to the administration of either BKM120+Anastrozole or BKM120+Docetaxel (Figure 5C, left panel). On the other hand, iTAp63 sensitizes basal BCSCs to the above-mentioned single and combinatorial therapeutic regimens (Figure 5C, left panel). We then analyzed ΔNp63 and TAp63 expression in the untrasduced BCSCs survived to the anti-tumor treatments. Interestingly, whereas TA expression levels were not markedly modulated upon treatments in both BCSC subtypes, ΔNp63 expression was significally up-regulated in basal BCSCs (Figure 5D), suggesting an involvement of ΔNp63 in drug resistance at least in this molecular subtype. Gene expression profile of a panel of effectors commonly involved in drug resistance/susceptibility revealed that iΔNp63 luminal cells showed high expression levels of drug resistance-related markers such as *FOS*, *EGFR*, *CYP2C8*, *CYP3A5* and *FGF2* (Figure 5E). Conversely, basal iTAp63 BCSCs showed an enrichment in *CDKN1A* (p21) [38] and *ESR1* (encoding for the estrogen receptor-alpha, ER-α) (Figure 5E). These data clearly suggest that TAp63 isoform plays a fundamental role in making aggressive breast cancer phenotypes more sensitive to hormone therapy, likely making them dependent on estrogen pathway through the up-regulation of ER-α in basal cancer. Significant change in ER-α expression was not observed in luminal BCSCs over-expressing ΔNp63 (Suppl. Figure 2G), indicating that cell refractoriness as well as tumorigenic and metastatic potential acquired by these cells is independent on *ESR1* expression levels. Moreover, in luminal BCSCs over-expressing ΔNp63 the silencing of PI3K (iΔNp63/shPI3K) hampered their invasion capacity whereas the invasive potential of basal TAp63 BCSCs was restored upon PI3K over-expression (iTAp63/PI3K) (Figure 5F). Of note, PI3K silencing highly reduced the expression of CD44v6 overcoming the effect of ΔNp63 (Figure 5G). These data confirmed that p63 isoforms orchestrate breast cancer progression through the activation of PI3K/CD44v6 axis (Suppl. Figure 3).

## DISCUSSION

The presence, role and characterization of the cells with tumorigenic and metastatic potential comprised in breast cancer is a matter of intense research [39, 40]. Here, we show that BCSCs, which express ΔNp63, are endowed with an intrinsic heightened invasive and metastatic activity that can be reverted to a phenotype with abrogated invasive potential by switching to the TAp63 expression.

Different groups have shown a moderate to intense p63 nuclear expression exclusively in the mammary gland's basal layer (where stem cells are located), with no detectable expression in the luminal cells (containing progenitor and differentiated cells) that line the glandular lumen [11]. In particular, basal cells were found enriched with ΔNp63, in contrast with the TAp63 expression that was restricted to the luminal epithelial glandular cells [10]. According to the current model, which describes CICs as derived from normal stem cells, it was shown that in tumor tissues, ΔNp63 was almost absent and confined to only some myoepithelial and basal cells, whilst most of the cells express the TAp63 protein. Different models were recently suggested with regard to ΔNp63 role in breast tumor progression. It was recently reported that ΔNp63 controls breast cancer cells migration and invasion [30, 41] and increases their tumor initiating activity [28, 29].

Our results extend these findings identifying a novel pathway regulated by p63 isoforms and highlighting also a role for the TAp63 isoforms.

Our findings show that BCSCs appear to express both TA and ΔNp63 and that ΔNp63 expression is lost when cancer cells develop a more differentiated phenotype. As expected ΔNp63 expressing cells have a more stem phenotype and appear to have a higher tumorigenic and metastatic potential.

Different studies in recent years have shown the ΔNp63 key role in the induction of EMT in breast cancers [42]. EMT has been described as a crucial step in tumor initiation and acquisition of metastatic traits [33, 43]. In line with these findings, we observe that ΔNp63 enhances the clonogenic potential and sustains an EMT programme mainly inducing the expression of *TWIST* and *PDGFRB*. The ability of TAp63 to delay the growth of primary tumors and abrogate metastasis formation, improving the survival rate of mice bearing tumors, may be related to the promotion of an epithelial phenotype observed in basal TAp63 BCSCs. The weak tumorigenic potential of luminal BCSCs could be attributed to the presence of a small cell subset expressing ΔNp63.

We also show that TA and ΔNp63 isoforms exert their opposite effects at least in part through the opposite regulation of a PI3K/CD44v6 pathway. Indeed, while ΔNp63 expression results in activation of the PI3K/AKT pathway leading to CD44v6 up-regulation and a more aggressive mesenchymal phenotype, TA expression has opposite effects. Blocking PI3K activation by means of inhibitors or silencing of CD44v6 reduces the ability of ΔNp63 to induce a more tumorigenic and invasive phenotype demonstrating the importance of this pathway. Clearly as reported in the literature [28–30, 41] this is not the only pathway controlled by p63 that appears more and more to be a master gene regulating cancer cell differentiation as it does in normal epithelial cells. Our data also show that the subset of cells that over-expresses ΔNp63 (presumably the cancer initiating subpopulation) are also as expected more resistant to current therapies but can be sensitized using PI3K inhibitors, suggesting that the use of these inhibitors in combination with currently used therapies could be an effective strategy to overcome resistance, prevent relapse and metastatic spreading.

## MATERIALS AND METHODS

### Tissue collection, isolation and culture of cancer cells

Breast cancer tissues were collected at the Department of Surgical and Oncological Sciences in accordance with the ethical standards of the University of Palermo institutional committee, and then characterized by tumor type, grading, or ER/PR/HER-2/KI67/p63 expression. Normal breast tissue was obtained from the histologically uninvolved resection. Breast tissues were mechanically and enzymatically digested using collagenase (1.5 mg/mL; Gibco) and hyaluronidase (20 mg/mL; Sigma Aldrich) in Dulbecco's Modified Eagle Medium (Gibco), for 1 hour at 37°C. Cells were maintained in culture in serum-free medium (SFM), in presence of fibroblast growth factor (bFGF; 10 ng/mL) and epidermal growth factor (EGF, 20 ng/mL) in ultra-low attachment flasks (Corning, Lowell, MA) as previously described [44]. These culture conditions make cells growing as aggregates conventionally defined as spheres. Weekly, spheres are enzymatically dissociated with accutase (Gibco) and plated in fresh SFM. The *in vitro* model adopted to obtain the non tumorigenic sphere-derived adherent cells (SDACs), is based on dissociation of BCSCs and subsequent culture in Dulbecco's modified Eagle medium (DMEM) supplemented with 10% FCS in adherent conditions for at least 25 days. Cancer associated fibroblasts (CAFs) were isolated, by enzymatic digestion, from basal breast cancer samples and cultured in presence of DMEM plus 10% FBS in adherent conditions.

### Cell proliferation

Cell proliferation was assessed using CellTiter 96^®^ AQueous One Solution Cell Proliferation Assay kit (MTS) according to the manufacturer's instruction.

The *in vitro* sensitivity assay was performed by plating 250 cells in ultra-low attachment 96-well plates (Corning) and exposing them to vehicle, 50 μM Anastrozole (Selleckchem), or 100nM Docetaxel (Selleckchem), alone or in combination with 5 μM BKM120 (NVP-BKM120, Selleckchem). Cell viability was measured for up to 72 hrs, using the CellTiter-Glo^®^ Luminescent Cell Viability Assay kit (Promega) according to the manufacturer's instruction. The results were analyzed by using Infinite^®^ F500 (Tecan). Each combinatorial regimen was evaluated in triplicate.

### Clonogenenic assay

The clonogenic capacity of BCSCs was assayed by dissociating breast cancer spheroids and plating them 1 cell/well in ultra-low attachment 96 well microplates with flat bottom (Corning, Lowell, MA), using a FACS Aria I. Results were statistically evaluated after 4 weeks.

### Migration and invasion assay

Transduced and control BCSCs were seeded in adherent conditions in six-well plates at a density of 1×10^6^ cells/well and cultured to achieve a confluent cell layer in culture medium. The day after a sterile 200 μl pipette tip was used to straight scratch a constant-diameter stripe in the confluent cell monolayer. Plates were then washed with 1 ml of PBS to remove detached cells and debris and the medium was replaced using a fresh culture medium. Wound healing was monitored, by EVOS™ fl Digital Inverted Fluorescence Microscope, following the migrating cells in the gap during a period of 24 hours post-scratch.

Cells tested for invasive potential (2×10^3^) were plated into Matrigel-coated (BD Biosciences) transwells of 8 μm pore size (Corning). DMEM supplemented with 10% human serum (Euroclone, ECS0219D) (600 μl/well) was used as chemo-attractant in the lower part of transwells. Migration was calculated by the microscopic observation of cells migrated in the lower part of transwell up to 72 hrs.

### Flow cytometry/cell sorting

Cells were stained with conjugated CD44-PE (G44-26, mouse, IgG2b, BD), CD24-APC (ML5, mouse, IgG2a, R&D), CD44v6-APC (2F10, mouse, IgG1, R&D) antibodies or matching isotype controls. Samples were acquired and analyzed by Accuri (BD Biosciences) flow cytometer. FACS sorting, using a FACS aria (BD Biosciences), was performed on cells stained with CD44-PE and CD24-APC, whose quality was monitored by flow cytometry. ALDH activity was measured by using ALDEFLUOR assay (Stem Cell technologies). All data were analyzed using FlowJo software (Tree Star). Sorted cells were collected and used for ΔNp63 and TAp63 mRNA expression.

### Immunohistochemistry/Immunofluorescence

For antigen unmasking, five-μm-thick paraffin-embedded sections of basal and luminal breast cancer tissues, such as their normal counterpart, were heated with a retrieval solution in 10 mM sodium citrate (pH 6.0). After rising in dH_2_O, endogenous peroxidase was inhibited by incubation of 5 minutes with 3% H_2_O_2_. Cells were permeabilized with PBS plus 0.1 % Triton X-100 (TBS) for 10 minutes on ice. Then cells were exposed to specific antibodies directed against ER (6F11, mouse IgG1 Leica), PR (1E2, rabbit IgG, Roche), HER-2 (D8F12, rabbit IgG, CST), CK5 (XM26, mouse IgG_1k_, Leica), CK8-18 (5D3, mouse IgG_1_, Leica), ALDH1 (44/ALDH, mouse IgG_1_, BD), p63 (4A4, mouse IgG_2a_, Santa Cruz), CK-AE (AE1/AE3, mouse IgG_1_, Novocastra), or isotype matched controls at appropriate dilutions overnight at 4°C. Sections were then incubated with biotinylated immunoglobulins, washed and following exposed to streptavidin. Stainings were revealed using 3-amino-9-ethylcarbanzole (AEC, Dako) substrate and counterstained with aqueous hematoxylin.

H&E staining was performed using standard protocols on 5-μm paraffin sections.

Immunofluorescence was performed on cytospun cancer sphere cells or SDACs cultured on round coverslips into 24 well plates, fixed with 2% paraformaldehyde for 20 minutes at 37°C or on five-μm-thick paraffin-embedded sections. After permeabilization, cells were stained overnight at 4°C using antibodies against ER, PR, HER-2, CK5, CK8-18, CK14, MUC1 (HMPV, mouse IgG_1k_, BD Biosciences), CD10 (FR4D11, mouse IgG_1_, Santa Cruz), VIMENTIN (R28, rabbit, CST), p63, GFP (D5.1, rabbit IgG, CST), RFP (ab62341, rabbit IgG, Abcam), or isotype-matched controls at appropriate dilutions. Then, cells were labelled with Alexa Fluor 488- or Rhodamine red-conjugated secondary antibodies (Thermo Fisher Scientific). Nucleus counterstaining was performed using Toto-3 iodide (RNAse treatment was performed to prevent the cytoplasmic RNA staining). Samples were analyzed using a confocal microscope (Nikon D-Eclipse C1).

### RNA extraction and real-time PCR

Breast cancer tissues and BCSCs were used to isolate RNA using the RNeasy Plus Mini Kit (Qiagen). Total RNA was retro-transcribed using the High-Capacity c-DNA Reverse Transcription kit (Applied Biosystem). Quantitative real-time PCR analysis was performed using a SYBR Green master mix containing the following primers: *TAp63* F-ATTGTTCTCCGTTCGTTG; *TAp63* R-GATGTAAGGGTCAGGGCAG; *ΔNp63* F-TATTGTAAGGGTCTCGGG; *ΔNp63* R-GGCATTGTTTTCCAGGTA; GAPDH F-GCTTCGCTCTCTGCTCCTCCTGT; *GAPDH* R-TACGACCAAATCCGTTGACTCCG. The Taqman master mix was prepared with the following primers: *CD44v6* Hs_01075870; *PI3K* Hs00907954_m1; *GAPDH* 4352934-1107035 (Applied Biosystem). The relative quantitation of gene expression was calculated on triplicate reactions using the comparative Ct method (ΔΔCt). *GAPDH* was used as housekeeping gene.

For the gene expression arrays, RNA samples were retro-transcribed (RT^2^ First Strand Kit, Qiagen) and real time PCR were performed using the RT^2^ Profiler PCR Arrays Human Epithelial to Mesenchymal Transition (PAHS-090ZR) and Human Cancer Drug Resistance (PAHS-004ZR). Collected data were analyzed with the Qiagen RT^2^ Profiler PCR Arrays data analysis software.

### Western blot

BCSCs were collected, washed twice in ice-cold PBS and then resuspended in ice-cold buffer containing TPER Reagent (Pierce) 300 mM NaCl (Sigma Aldrich) 1 mM orthovanadate (Sigma Aldrich) 2 mM pefabloc (Roche), proteinase inhibitor cocktail (aprotinin, pepstatin and leupeptin, each at 5 μg/mL, Sigma Aldrich) and incubated for 30 min on ice. Protein extracts were resolved using a SDS-PAGE gel and separated by electrophoresis, transferred to nitrocellulose membranes (Hybond-C Extra, Nitrocellulose, Amersham) and subsequently blocked with PBS Tween 20 0.1% (Sigma Aldrich) not-fat dry milk 5% (Sigma-Aldrich) for 1 hour at R.T. Immunodetection was performed by incubating the membranes with the primary antibodies at 4°C O.N., and finally with secondary antibodies for 1 hour at R.T. The following antibodies were used: p63-α (D2K8X, rabbit IgG, CST), p63 (4A4, mouse IgG2A, Santacruz), p110-α (PI3K, C73F8, rabbit IgG, CST), AKT (rabbit, CST #9272), P-AKT (D9E, rabbit, IgG, CST), β-ACTIN (8H10D10, mouse IgG_2b_, CST), anti-mouse HRP-conjugated (goat IgG, ThermoFisher Scientific), anti-rabbit HRP-conjugated (goat IgG, ThermoFisher Scientific). Immunoreactive bands were detected using SuperSignal™ West Dura Extended Duration Substrate (ThermoFisher Scientific) and revealed by using the Amersham imager 600 (GE Healthcare).

### Production of lentiviral particles and BCSCs transduction

Gene transfer of luciferase (LUC) and green fluorescent protein (GFP) was assessed using the p-TWEEN lentiviral vector. The synthetic genes encoding ΔNp63-α (Addgene #26979) and TAp63-γ (Addgene #26977) were inserted into the p-TWEEN EGFP and p-TWEEN RFP lentiviral expression vector, and in the pInducer20 Tet-inducible lentiviral vector (kindly provided by J.P. Medema). The synthetic gene encoding CD44v6 (Eurofins MWG Operon) and PI3KCA cDNA (Addgene, 12523) were inserted into the pTWEEN EGFP expression vector.

shp63 (GTCATTTGATTCGAGTAGA) and scramble sequences were cloned in a lentiviral Lentilox 3.7 vector. shCD44v6 (ACAGATGGCATGAGGGATATC), shPI3K (GCGAAATTCTCACACTATTAT) and scramble sequences were cloned downstream U6 promoter of the lentiviral pLK0.1 vector.

Lentiviral supernatants were collected 48 hours after transfection of the packaging cell line HEK-293T. Transfection was performed by using X-treme GENE HP DNA Transfection Reagent (Roche). Cells (1 × 10^5^) were then transduced with 1 ml of viral supernatant for 24-48 hours using 8μg/mL polybrene. All the experiments using transduced cells, started 5 days after the transduction. For the double transduction of inducible (iTAp63 and and iΔNp63) and over-espressing/down-regulating CD44v6 (v6 and shv6) vectors, BCSCs were firstly transduced with iEV, iTAP63 and iΔNp63, and after one week with vectors coding for CD44v6 (v6) or shCD44v6 (shv6) and their controls (EV and Scr). After 7 days, doxycycline was added (to induce expression of the p63 isoforms) for 5 days.

### Cytokines quantification and cell treatment

CAFs conditioned medium was derived from cells plated at approximately 70-80% of confluence in T25 flasks and grown for 48-hours in 10 ml serum-free stem cell medium with 50 mg/mL ascorbic acid. Quantification of HGF, SDF-1 and OPN production of BCSCs or CAFs was measured using multiplex Bio-Plex Pro Assays (Bio-Rad). Raw data (mean fluorescence intensity) from all kits were analyzed by Bio-Plex Software (Bio-Rad).

For cytokines treatment, BCSCs were starved of EGF and b-FGF overnight and then treated for 24 hours with HGF (100 ng/ml), SDF-1 (100ng/ml), OPN (1 μg/ml), or CAF-CM. Treated cells were collected and used for RNA extraction.

### Animals and tumor models

Mice experiments were performed according to the animal care committee guidelines of the University of Palermo. All the *in vivo* experiments were performed in 6 mice per condition using the 2 more tumorigenic luminal (patient #4 and #18, see Table 1) and 2 basal (patient #10 and #30) BCSCs. For the tumorigenic assay cells were harvested, suspended in 100 μl of 1:1 Matrigel (BD Matrigel Matrix Growth Factor Reduced) and then injected subcutaneously into 5-week-old NOD/SCID mice (Charles River Laboratories, Milan, Italy). To test the invasive capacity of luminal BCSCs over-expressing ΔNp63, a 1:1 mixture of EV-GFP:EV-RFP or TAp63-GFP:ΔNp63-RFP transduced cells was suspended in Matrigel and subcutaneously injected in NOD/SCID mice. Tumor growth was followed over time after cell injection and their size was measured weekly using an electronic caliper. The volume was then calculated with the formula: largest measured diameter × (smallest measured diameter)^2^ × π/6. Serial passages of tumors were performed by injecting 4,000 BCSCs subcutaneously in 100 μl of 1:1 Matrigel. Sub-renal capsule injections were performed using 4,000 cells transduced with LUC/GFP in 50 μl of 1:3 Matrigel. Tumor and metastasis formation was monitored over time and tracked *in vivo* by using the Photon IMAGER (Biospace Lab) for up to 14 weeks. At the endpoints, mice were sacrificed following IACUC guidelines, and xenograft tumor samples collected. Xenograft tumor tissues were collected and used for paraffin-embedded tissues, RNA, protein, and cell isolation. The survival rate was evaluated on the basis of photon emission, in particular the mice were sacrificed when the total amount of photons/s/sr of all secondary lesions was 4×10^3^, which corresponded to a ≤0.5 cm^2^ tumor metastatic area.

### Statistical analysis

Results are shown as the mean ± SD for at least three repeated independent experiments for each group. The mean and SD were obtained by analyzing replicates using Prism 5 (GraphPad Software, La Jolla, CA, USA) applying two-tails Student's *t*-test for independent experiments. Significance levels were indicated as *P* values. * indicates *P* < 0.05, ** indicate *P* < 0.01 and *** indicate *P* < 0.001. ns indicates non statistically significant.

## SUPPLEMENTARY MATERIALS


