# Supplementary material for: ΔNp63 drives metastasis in breast cancer cells via PI3K/CD44v6 axis
Source: Oncotarget. 2016 Aug 2;7(34):54157–73. doi: 10.18632/oncotarget.11022 (PMC5342332; doi:10.18632/oncotarget.11022)
Supplement: Supplementary file 1 [file oncotarget-07-54157-s001.pdf]

ΔNp63 drives metastasis in breast cancer cells *via* PI3K/CD44v6 axis

Supplementary Material

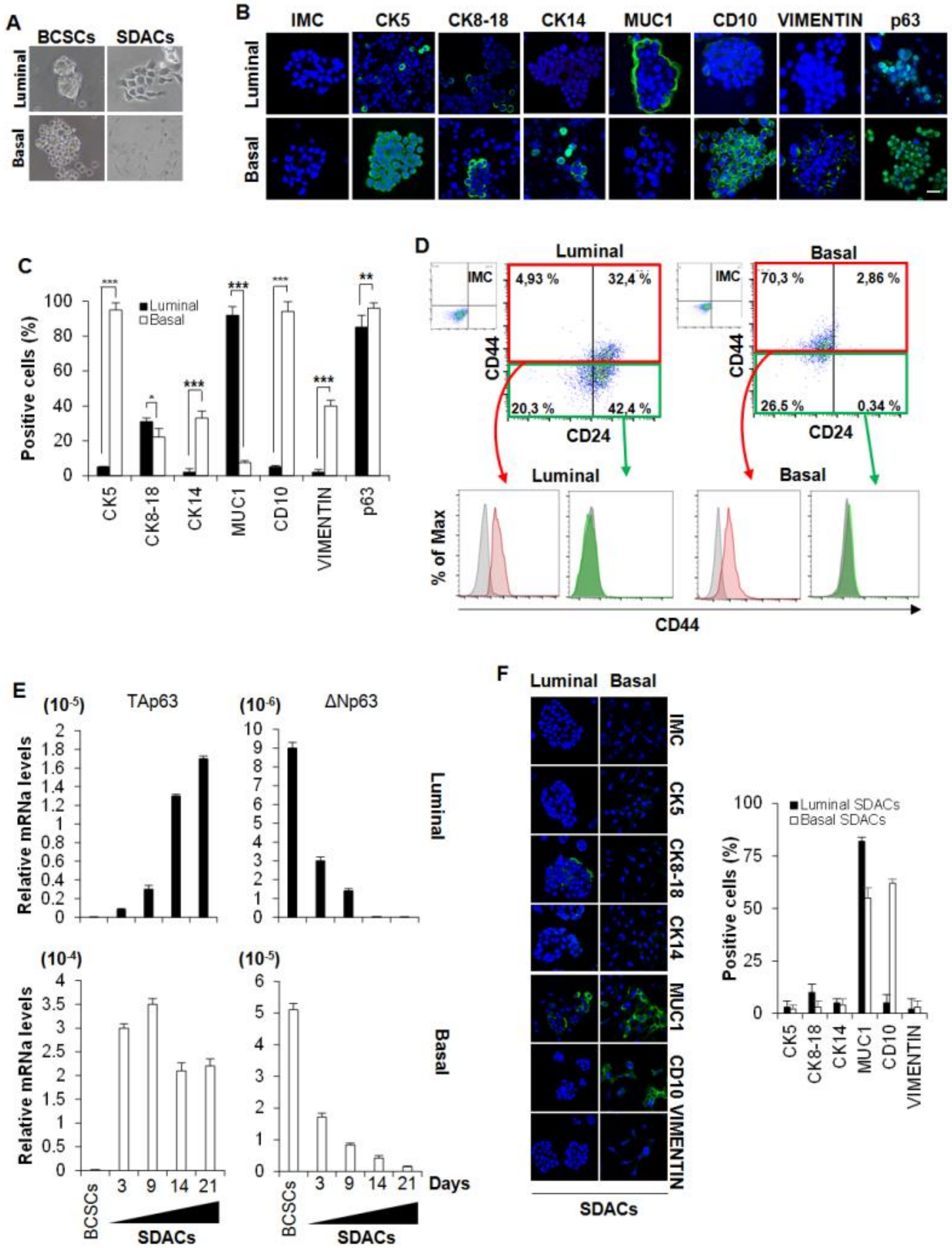

Figure S1. Basal BCSCs express high levels of CD44

(A) Representative phase contrast microscopic analysis of luminal and basal BCSCs and their derived SDACs. (B) Representative immunofluorescence analysis of CK5, CK8-18, CK14, MUC1, CD10, VIMENTIN and p63 of 3 luminal and 2 basal BCSCs cytospins. Scale bar represents 20 μm. (C) Percentage of CK5, CK8-18, CK14, MUC1, CD10, VIMENTIN and p63 positivity as in (B). Data are expressed as mean ± SD of 3 independent experiments performed in 3 luminal and 2 basal patient-derived BCSC lines. Positivity was calculated by confocal microscopy examination of three independent observers. (D) (Upper panels) Representative flow cytometry expression profiles of CD44 and CD24 of 3 luminal and 2 basal BCSCs. (Lower panels) Post-sorting analysis of CD44 expression in enriched $CD44^+$ and $CD44^-$ luminal and basal BCSCs. Grey histograms show the isotype matched controls. (E) Relative *TAp63* and *ΔNp63* expression in luminal and basal SDACs at the given time points. Data are expressed as mean ± SD of 3 independent experiments performed in 3 luminal and 2 basal BCSC lines. (F) Immunofluorescence analysis (left panels) and percentage of positivity (right panel) for CK5, CK8-18, CK14, MUC1, CD10, VIMENTIN performed in luminal and basal SDACs. Data are expressed as mean ± SD of 3 independent experiments performed in 3 luminal and 2 basal patient-derived BCSC lines. * indicates P<0.05, ** indicate P<0.01 and *** indicate P<0.001. ns indicates non statistically significant.

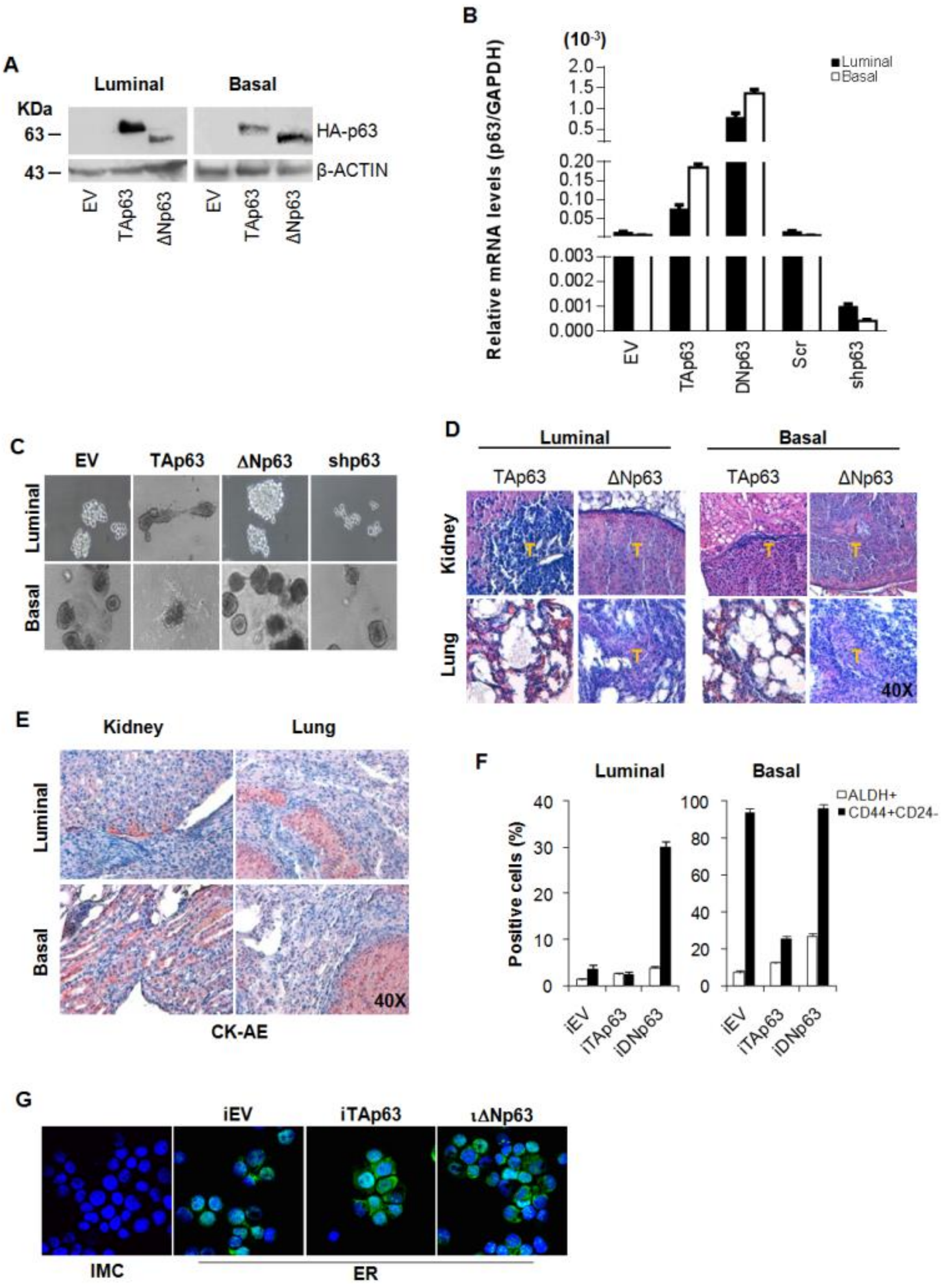

Figure S2. BCSCs overexpressing ΔNp63 retain clonogenic and metastatic potential

(A) Immunoblot analysis of luminal and basal BCSCs transduced with empty vector (EV) or with lentivirus hemagglutinin TAp63 or ΔNp63 (HA)-tagged. β-ACTIN was used as loading control. (B) *p63* mRNA expression levels in luminal and basal BCSCs transduced with lentiviral vector encoding for empty vector (EV), TAp63, ΔNp63, scrambled (Scr) or

p63 shRNA (shp63) sequences. *GAPDH* amplification was used as endogenous control. Results show mean ± SD of 3 independent experiments performed in 3 luminal and 2 basal BCSC primary lines. (C) Phase contrast microscopic analysis of luminal and basal BCSCs over-expressing TAp63, ΔNp63 or p63 shRNA (shp63). Empty vector (EV) was used as transduction control. (D) Haematoxylin and eosin (H&E) performed on paraffin embedded sections of sub-renal capsule tumors and their metastasis generated by injection of 4,000 TAp63 and ΔNp63 BCSCs. T refers to tumor area. (E) Immunohistochemical analysis of human CK-AE on tumors and metastasis as in (D). (F) Flow cytometry expression profiles of CD44, CD24 and ALDH activity ($ALDH^{+}$) of 3 luminal and 2 basal BCSCs transduced with iEV, iTAp63 and iΔNp63. (G) Immunofluorescence analysis for ER on luminal BCSCs transduced with iEV, iTAp63 and iΔNp63.

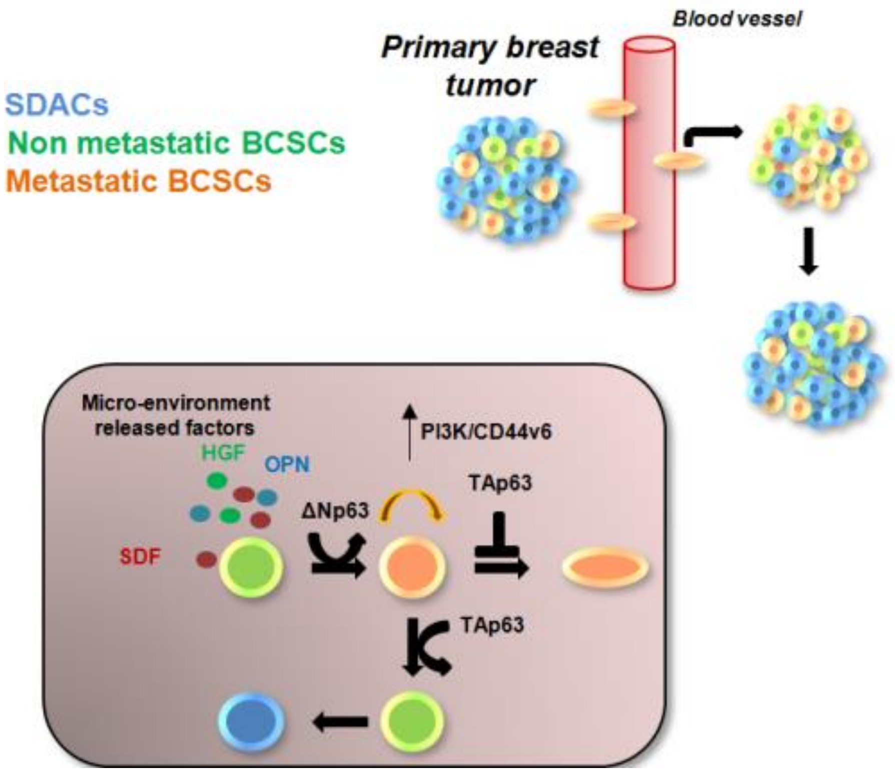

Figure S3. ΔNp63 induces CD44v6 regulation by PI3K activation

Schematic model of p63 isoforms involvement in driving breast cancer progression.
